# Complete mitochondrial genome of a golden orb-web spider *Trichonephila clavata* (Chelicerata, Arachnida) from South Korea

**DOI:** 10.1080/23802359.2021.1955633

**Published:** 2023-07-04

**Authors:** Eun Hwa Choi, Ui Wook Hwang

**Affiliations:** a Department of Biology Education, Teachers College and Institute for Phylogenomics and Evolution, Kyungpook National University, Daegu, South Korea; b Institute for Korean Herb-Bio Convergence Promotion, Kyungpook National University, Daegu, South Korea; c School of Industrial Technology Advances, Kyungpook National University, Daegu, South Korea

**Keywords:** *Trichonephila clavata*, Nephilinae, Chelicerata, mitochondrial genome, phylogeny

## Abstract

The mitochondrial genome of a golden orb-web spider *Trichonephila clavata* (L. Koch, 1878) from South Korea is determined and characterized in detail, which is the second mitochondrial genome reported from this species: the first was published from the Chinese sample by Pan et al. (2016). It was 14,436 bp in length being composed of 13 protein-coding genes (PCGs), 22 transfer RNA genes, two ribosomal RNA genes, and one control region (CR). It has a base composition of 35.99% for ‘A,’ 14.88% for ‘G,’ 9.09% for ‘C,’ and 40.04% for ‘T.’ Comparing the South Korean and Chinese mitochondrial genomes, we observed 8% nucleotide sequence differences between their CRs, caused by the different numbers and sorts of possessed tandem repeats, suggesting a promising molecular marker to distinguish South Korean individuals from Chinese ones. The phylogenetic trees using the maximum likelihood (ML) method were reconstructed with nucleotides (without 3rd codon position) and amino acids from 13 PCGs, respectively, which consistently confirmed that *T. clavata* (Subfamily Nephilinae) from South Korea and China are clustered together, distinctly separated from the other subfamily Araneinae in the monophyletic family Araneidae.

The golden orb-web spiders of the genus *Nephila* are tropical and subtropical in distribution and construct exceptionally large and impressive orb webs (Kuntner et al. 2010, 2013). *Nephila clavata* L. Koch, 1878 native to East Asia is distributed ranging from India to Japan in the World Spider Catalog version 22.0 database (Natural History Museum Bern 2021). Recently, combined with molecular information including six genes (three mitochondrial and three nuclear genes), these results have led to a comprehensive phylogenetic classification (Wheeler et al. 2017). It has been reported that the family Araneidae including *N. clavata*, one of the nepheline group is well-supported with a bootstrap value of 83%. Since 2019, *N. clavata* has been moved from the genus *Nephila* into the genus *Trichonephila*, along with other 11 *Nephila* species (Kuntner et al. 2019). Currently, *Trichonephila clavata* (L. Koch, 1878) is taxonomically acceptable. From the species, Pan et al. (2016) first reported the complete mitochondrial genome from a Chinese sample of *T*. *clavata*, but mitochondrial genome information from South Korean individuals has not been investigated so far.

Here, we collected a specimen of *T*. *clavata* from the Daegu campus of Kyungpook National University (KNU; 35°53′13.7″N 128°36′21.1″E), which was stored in 95% ethanol and deposited under the voucher number KNU-ARAR-001 at KNU (collector: Ui Wook Hwang, uwhwang1@gmail.com). Total cellular DNA was extracted from a spider leg using a DNeasy tissue kit (QIAGEN Co.) following the manufacturer’s instructions. First, we amplified the partial region (465 bp long) of *16S rRNA* with universal PCR primers: 16SA (5′-CGCCTGTTTATCAAAAACAT − 3′; Simon et al. 1994) and 16SB (5′-CCGGTTGAACTCAGATCA − 3′; Kambhampati and Smith 1995). Then long PCR amplification of the complete mitochondrial genome for *T. clavata* was carried out with a pair of species-specific long PCR primers designed from the sequenced *16S rRNA* (refer to Hwang et al. 2001): Long Spi (+) 5′-ACATGGAGCAGGTTTTACTAATAATTTAAGAAGA − 3′ and Long Spi (-) 5′-AGTTCATATTAAAAAAAAAGATTGCGACCTCGAT − 3′. The long PCR products were sequenced using an ABI PRISM 3730 sequencer (Macrogen Co., South Korea). 13 PCGs and 2 rRNA genes were identified through BLAST searches and alignment with known genes using Clustal X2 (Larkin et al. 2007). Twelve tRNA genes were detected using tRNAscan-SE (Chan and Lowe 2019), and the remaining 10 tRNAs were identified by eye based on their potential for forming tRNA-like secondary structure. For the subsequent phylogenetic analyses, we constructed two concatenated sequence alignment sets from the 13 PCGs from 19 spider species: nucleotide sequence alignment set (6931 bp without 3rd codon position) and amino acid sequence alignment set (3387 aa). Maximum likelihood (ML) trees using IQ-TREE webserver (Trifinopoulos et al. 2016) were reconstructed under the best-fit substation models: GTR + F + I + G4 and mtART + F+I + G4 for the nucleotide and amino acid sequence substitution models in order. Node confidence values in percent were estimated with 1000 bootstrap replicates.

The entire mitochondrial genome of *T. clavata*, which is 14,436 bp in length (GenBank accession no. NC_008063), consists of 37 genes including 13 PCGs, two rRNA genes, and 22 tRNA genes, with a control region (CR). The overall base composition is 35.99% for ‘A,’ 9.09% for ‘C,’ 14.88% for ‘G,’ and 40.04% for ‘T,’ showing strong A + T biased composition (76.03%). Among 13 PCGs, ‘ATA’ as a start codon was used for *ND1*, *ND2*, *ND4*, *ND5*, *CYTB*, and *ATP6*, ‘ATT’ for *ND3*, *ND4L*, *ND6*, and *ATP8*, ‘TTG’ for *COX2* and *COX3*, and ‘TTA’ for only *COX1*. As stop codons, ‘TAA’ was used for *COX1*, *COX2*, *ND3*, *ND4*, *ND4L*, *ND5*, *ATP6*, and *ATP8*, ‘TAG’ for *ND1*, *ND2*, *ND6*, and *CYTB*, and ‘T’ for *COX3*. When we compared the present result with the mitochondrial genome published from the same species of China (Pan et al. 2016), we observed the highest sequence differences only in CR (ca. 8%), caused by the different numbers and sorts of possessed tandem repeats: 11 and 9 tandem repeats of 5′-GATATATACATATATA − 3′ for the South Korean and Chinese ones, respectively, and 5 additional tandem repeats of 5′-TCTATACATATATA − 3′ only for the Chinese one. The result implies that CR can be a useful molecular marker for distinguishing South Korean individuals from Chinese ones (e.g., like in a species identification tool in *Mesobuthus* reported by Choi et al. 2007).

To determine the phylogenetic position of *T. clavata* in the family Araneidae, we constructed both the concatenated nucleotide sequence alignment without 3rd codon position (NT) and the concatenated amino acid sequence alignment (AA) from 19 araneid species (Hwang and Kim 1999). We employed *Tetragnatha nitens* and *Tetragnatha maxillosa* as outgroups. As shown in Figure 1, the ML trees based on NT and AA yielded almost same topology, but the former was more resolved and reliable than the latter. In the trees, major clades are continuously supported: the monophylies of the family Araneidae (BP 100% in NT/BP 100% in AA) and the subfamily Araneinae (BP 99%/BP 100%). *T. clavata* from South Korea and China, which are only members of the subfamily Nephilinae examined in this study, were clustered together with a high nodal support (BP 100%/BP 100%), and distinctly separated from the monophyletic clade of Araneinae.

**Figure 1.**
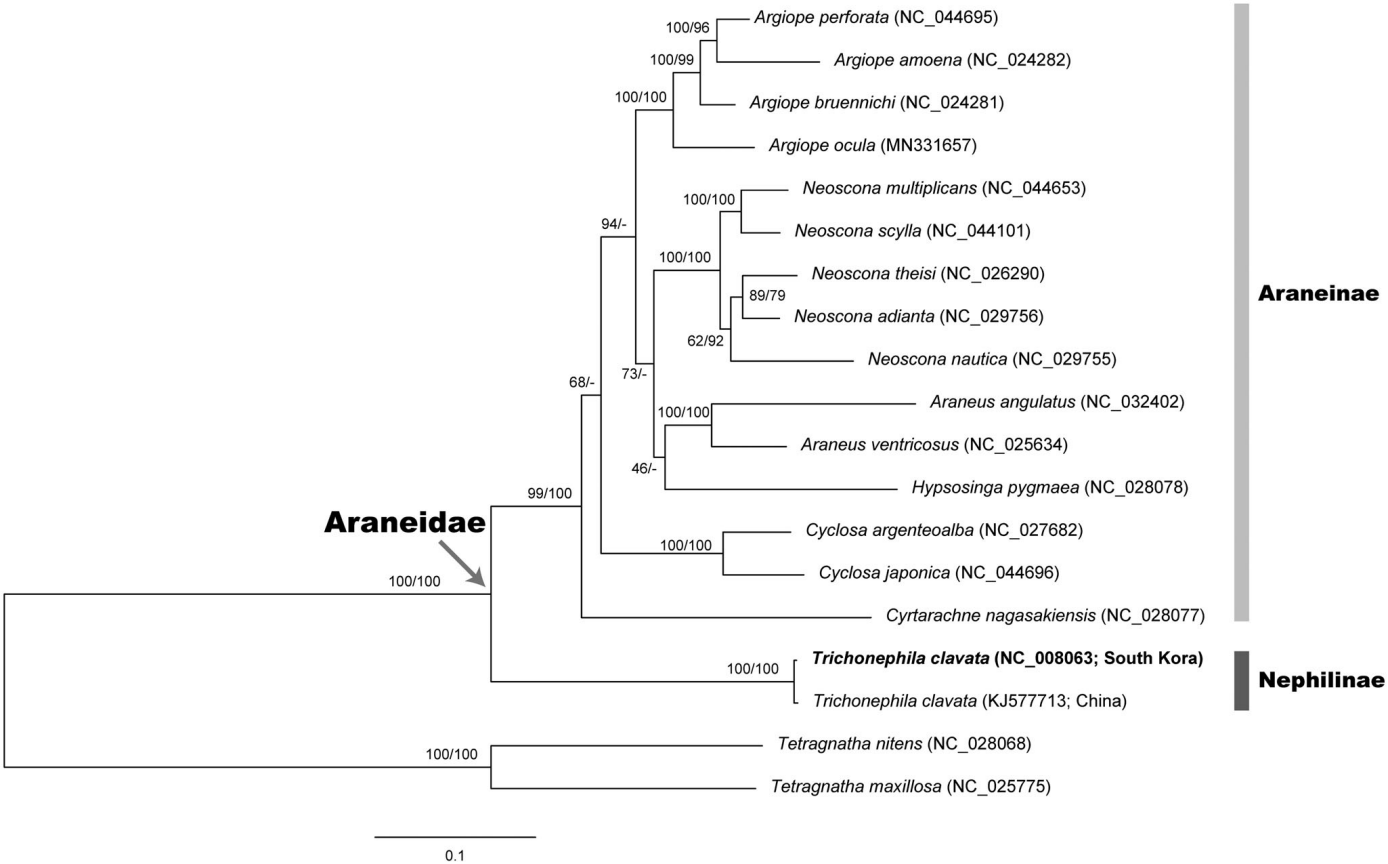
A maximum-likelihood (ML) tree reconstructed with the nucleotide sequence alignment set (without 3rd codon position) of 13 mitochondrial protein-coding genes (PCGs) showing relationships among 19 spider species belonging to the family Araneidae. Two tetragnathid spider species *Tetragnatha nitens* and *Tetragnatha maxillosa* are used as outgroups. The ML tree based on amino acid sequence alignment set from 13 PCGs yielded almost same topology with the nucleotide-based ML tree. Nodal supports are inferred from the ultrafast bootstrap method with 1000 bootstrap replicates using the IQ-TREE webserver: bootstrapping values in percent (BP) obtained using the nucleotide data set (left) and amino acid data set (right) on each node.

## Data Availability

The data that support the findings of this study are openly available in GenBank of NCBI at https://www.ncbi.nlm.nih.gov/nuccore/NC_008063.1.

## References

[CIT0001] Chan PP, Lowe TM. 2019. tRNAscan-SE: searching for tRNA genes in genomic sequences. Methods Mol Biol. 1962:1–14. 31020551 10.1007/978-1-4939-9173-0_1 PMC6768409

[CIT0002] Choi EH, Park SJ, Jang KH, Hwang UW. 2007. Complete mitochondrial genome of a Chinese scorpion *Mesobuthus martensii* (Chelicerata, Scorpiones, Buthidae). DNA Seq. 18(6):461–471. 17676475 10.1080/10425170701289883

[CIT0003] Hwang UW, Kim W. 1999. General properties and phylogenetic utilities of nuclear ribosomal DNA and mitochondrial DNA commonly used in molecular systematics. Korean J Parasitol. 37(4):215–228. 10634037 10.3347/kjp.1999.37.4.215 PMC2733198

[CIT0004] Hwang UW, Park CJ, Yong TS, Kim W. 2001. One-step PCR amplification of complete arthropod mitochondrial genomes. Mol Phylogenet Evol. 19 (3):345–352. 11399145 10.1006/mpev.2001.0940

[CIT0005] Kambhampati S, Smith PT. 1995. PCR primers for the amplification of four insect mitochondrial gene fragments. Insect Mol Biol. 4(4):233–236. 8825760 10.1111/j.1365-2583.1995.tb00028.x

[CIT0006] Kuntner M, Arnedo MA, Trontelj P, Lokovšek T, Agnarsson A. 2013. A molecular phylogeny of nephilid spiders: evolutionary history of a model lineage. Mol Phylogenet Evol. 69(3):961–979. 23811436 10.1016/j.ympev.2013.06.008

[CIT0007] Kuntner M, Gregorič M, Li D. 2010. Mass predicts web asymmetry in *Nephila* spiders. Naturwissenschaften. 97(12):1097–1105. 21060982 10.1007/s00114-010-0736-1

[CIT0008] Kuntner M, Hamilton CA, Cheng R-C, Gregorič M, Lupše N, Lokovšek T, Lemmon EM, Lemmon AR, Agnarsson I, Coddington JA, et al. 2019. Golden orbweavers ignore biological rules: Phylogenomic and comparative analyses unravel a complex evolution of sexual size dimorphism. Syst Biol. 68(4):555–572. 30517732 10.1093/sysbio/syy082 PMC6568015

[CIT0009] Larkin MA, Blackshields G, Brown NP, Chenna R, McGettigan PA, McWilliam H, Valentin F, Wallace IM, Wilm A, Lopez R, et al. 2007. Clustal W and Clustal X version 2.0. Bioinformatics. 23(21):2947–2948. 17846036 10.1093/bioinformatics/btm404

[CIT0010] Natural History Museum Bern. 2021. World spider catalog. Version 22.0. Online at http://wsc.nmbe.ch.

[CIT0011] Pan W-J, Fang H-Y, Zhang P, Pan H-C. 2016. The complete mitochondrial genome of *Nephila clavata* (Araneae: Nephilidae) Chinese population. Mitochondrial DNA Part A. 27(2):997–995. 10.3109/19401736.2014.926521 24938104

[CIT0012] Simon C, Frati F, Beckenbach A, Crespi B, Liu H, Flook P. 1994. Evolution, weighting and phylogenetic utility of mitochondrial gene sequences and a compilation of conserved polymerase chain reaction primers. Ann Entomol Soc Amer. 87(6):651–701.

[CIT0013] Trifinopoulos J, Nguyen LT, von Haeseler A, Minh BQ. 2016. W-IQ-TREE: a fast online phylogenetic tool for maximum likelihood analysis. Nucleic Acids Res. 44(W1):W232–W235. 27084950 10.1093/nar/gkw256 PMC4987875

[CIT0014] Wheeler WC, Coddington JA, Crowley LM, Dimitrov D, Goloboff PA, Griswold CE, Hormiga G, Prendini L, Ramírez MJ, Sierwald P, et al. 2017. The spider tree of life: phylogeny of Araneae based on target-gene analyses from an extensive taxon sampling. Cladistics. 33(6):574–616. 34724759 10.1111/cla.12182

